# Carbon-Rich Nanocomposites Based on Polyaniline/Titania Nanotubes Precursor: Synergistic Effect Between Surface Adsorption and Photocatalytic Activity

**DOI:** 10.3390/molecules30122628

**Published:** 2025-06-17

**Authors:** Brankica Gajić, Milica Milošević, Dejan Kepić, Gordana Ćirić-Marjanović, Zoran Šaponjić, Marija Radoičić

**Affiliations:** 1 “Vinča” Institute of Nuclear Sciences, National Institute of Republic of Serbia, University of Belgrade, Mike Petovića Alasa 12-14, 11000 Belgrade, Serbia; brankica.gajic@vin.bg.ac.rs (B.G.); milicam@vin.bg.ac.rs (M.M.); dejankepic@gmail.com (D.K.); 2 Faculty of Physical Chemistry, University of Belgrade, Studentski Trg 12-16, 11158 Belgrade, Serbia; gordana@ffh.bg.ac.rs; 3 Institute for General and Physical Chemistry, Studentski Trg 12-16, 11000 Belgrade, Serbia; zsaponjic@iofh.bg.ac.rs

**Keywords:** carbonized polyaniline, titania nanotubes, photocatalysis, carbon-rich materials, dye adsorption, environmental

## Abstract

Nowadays, there is an urgent need for efficient photocatalysts and adsorbents for environmentally relevant applications. This study investigates the effect of polyaniline (PANI) on the structure and performance of carbonized nanocomposites composed of PANI and TiO_2_ nanotubes (NTs), focusing on their photocatalytic degradation efficiency and dye adsorption capacity. The hypothesis was that PANI forms conductive carbon domains and stabilizes the anatase phase during thermal treatment, enhancing the performance of TiO_2_-NTs as photocatalysts. Nanocomposites based on PANI and TiO_2_-NTs (TTP) were synthesized through chemical oxidative polymerization of aniline (ANI) in the presence of TiO_2_-NTs using two TiO_2_/ANI molar ratios of 50 and 150 and subsequently carbonized at 650 °C, yielding CTTP-50 and CTTP-150. The novel CTTP composites and carbonized pristine TiO_2_-NTs (CTNT) were characterized by various techniques, including TEM, UV-Vis diffuse reflectance, Raman spectroscopy, XRD, and TGA. Their performance regarding dye adsorption and photocatalytic degradation under visible light was evaluated with Acid Orange 7, Methylene Blue, and Rhodamine B. CTTP-150 exhibited the highest adsorption capacity and photodegradation rate, attributed to the synergistic effect of PANI, which stabilizes the TiO_2_ phase and enhances visible-light absorption and adsorption.

## 1. Introduction

The rapid global expansion of the chemical and electronics industries has contributed to significant environmental problems [1]. The effective removal of organic pollutants from wastewater remains one of the most pressing problems in environmental chemistry. In this context, photocatalysis has emerged as a promising approach to combat pollution through the photodegradation of organic compounds [2,3,4]. Among various materials, titanium dioxide (TiO_2_, titania) has been widely used as a powerful photocatalyst. Both in the form of nanoparticles and nanotubes, it showed promising applications for the photocatalytic removal of pollutants (dyes, phenols, etc.) from aqueous media [5]. However, their effectiveness in environmental remediation is limited by their low efficiency in visible light and the rapid recombination of electron–hole pairs [6]. Titanium dioxide-based composites have attracted considerable attention in environmental research due to their favorable properties, including thermal stability, low cost, and non-toxicity [7,8,9]. Nevertheless, the wide band gap of TiO_2_ (3.2 eV), which limits its activity in visible light, is a significant obstacle to its wider industrial application [10,11]. Therefore, improving the light-collecting ability and extending the lifetime of the charge carriers generated by the light are crucial for enhancing the photocatalytic degradation of organic dyes with titania-based materials. Numerous modification strategies have been explored to overcome these limitations and enhance the photocatalytic performance of TiO_2_, e.g., doping and coupling in composites with other semiconductors, conductive polymers, carbon materials, etc. [6,12,13,14,15,16].

Polyaniline (PANI), a conductive polymer belonging to the class of intrinsically conductive polymers (ICPs), has attracted considerable attention in recent years due to its unique combination of electrical conductivity, environmental stability, and tunable optical properties [17,18]. These properties make PANI a promising candidate for improving the photocatalytic performance of TiO_2_, especially by using it as a photosensitizer in composite systems. Numerous studies have shown that the integration of PANI into TiO_2_ matrices can significantly improve the separation efficiency of photoinduced electron–hole pairs, extend the spectral response of TiO_2_ into the visible region, and ultimately increase the overall photocatalytic activity of the resulting hybrid material [17,19].

The development of PANI/TiO_2_ nanocomposites has therefore become an active area of research, particularly for applications in environmental remediation and solar energy conversion. Various synthesis techniques have been used to prepare neat PANI and these composites, including chemical oxidative polymerization (in situ or ex situ), sol–gel processing, and hydrothermal or solvothermal methods [12,20,21,22,23,24]. Each technique offers different advantages in terms of morphology control, surface functionalization, and interfacial contact between the TiO_2_ and PANI phases. Recent studies have also shown that the variation of these parameters can significantly affect the charge transfer at the interface, light absorption, and stability of the hybrid photocatalysts [18,20]. Notably, Galloni et al. demonstrated that the use of milder oxidative conditions leads to significant differences in the morphology, porosity, and conductivity of the resulting PANI materials, thereby impacting their suitability for various applications [25]. Although PANI/TiO_2_ systems are not new, developing carbonaceous PANI/TiO_2_ composites, particularly based on TiO_2_ nanotubes, remains completely unexplored. Our approach, which combines optimized oxidative polymerization with controlled carbonization, aims to overcome previous limitations and provide new insights into the structure–activity relationships of such systems.

In recent years, the carbonization of PANI has emerged as a promising strategy to further enhance the properties of PANI/TiO_2_-based nanocomposites [13]. Upon thermal treatment in an inert atmosphere, PANI undergoes carbonization, yielding nitrogen-doped carbonaceous structures with graphene-like characteristics, showing increased electrical conductivity and specific surface area compared to the original PANI [26,27,28,29]. These carbonized materials not only preserve the conductive nature of the polymer, but also introduce additional N- and O-containing functionalities, which can modulate the electronic structure of TiO_2_ in the resulting carbonized PANI/TiO_2_ composite by forming chemical bonds (e.g., Ti–N, Ti–C) or intimate carbon–semiconductor interfaces [30,31]. Such modifications promote more efficient charge transfer, suppress electron–hole recombination, and extend light absorption into the visible spectrum. Furthermore, the presence of carbonized PANI contributes to a higher surface area, enhanced structural stability, and improved dispersion of TiO_2_ nanoparticles, all of which are crucial factors for achieving superior photocatalytic performance [13].

Despite the extensive research in the field of PANI/TiO_2_ composites, specific considerations remain relevant for further development. Although the synthesis of polyaniline has been widely researched, it usually requires the use of oxidizing agents and acidic media, which can lead to environmental problems if not handled properly, especially in large-scale production processes [17]. The situation is similar with titanium dioxide, which is generally regarded as a stable and effective photocatalyst, but is classified in the literature as a possible Group 2B carcinogen by the International Agency for Research on Cancer (IARC) due to occupational exposure to ultrafine nanoparticles only through inhalation [32]. These factors highlight the importance of developing composite materials with improved safety profiles and reduced environmental impact, particularly through synthetic processes that allow better control over composition and structure.

In this study, we report the development of carbon-rich CTTP nanocomposites using a two-step method: oxidative polymerization of aniline in the presence of TiO_2_ nanotubes, followed by controlled thermal carbonization. This approach enables the formation of nitrogen-doped carbon domains that improve conductivity and structural integrity while minimizing the remaining reactive species. The synthesized CTTP nanocomposites were characterized in detail using Raman spectroscopy, X-ray diffraction (XRD), transmission electron microscopy (TEM), and UV–Vis diffuse reflectance spectroscopy (DRS). Three organic dyes—Acid Orange 7 (AO7), Methylene Blue (MB), and Rhodamine B (RB)—were selected as model pollutants to comprehensively evaluate the adsorptive and photocatalytic properties of the synthetically produced materials. These dyes were chosen because of their structural diversity, their industrial importance, and their different charge properties: AO7 is an anionic azo dye typically found in textile wastewater; MB is a cationic dye known for its strong adsorption via electrostatic and π–π interactions, while RB is a zwitterionic xanthene dye that exhibits both positive and negative charges under neutral pH conditions.

This work aims to contribute to the development of highly efficient photocatalytic and adsorbent materials for environmental remediation, with a focus on understanding the relationship between the chemical structure of dyes, the surface structure of the synthesized catalysts, and their photocatalytic and adsorption ability. To the best of our knowledge, this is the first report demonstrating the synergistic interplay between carbonized PANI and TiO_2_-NTs, leading to a simultaneous improvement in visible-light photocatalysis and dye adsorption efficiency. This work presents a unique and environmentally conscious contribution to the field of hybrid photocatalytic materials.

## 2. Results and Discussion

### 2.1. Morphological Properties of Synthesized Materials

**Figure 1:**
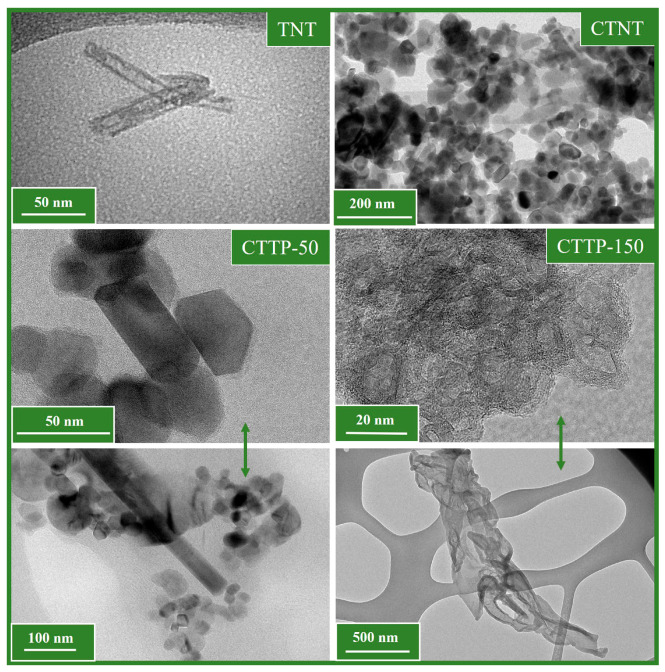
TEM images of pristine TiO_2_-NTs (TNT), annealed TiO_2_-NTs (CTNT), and carbonized PANI/TiO_2_-NT nanocomposite samples (CTTP-50 and CTTP-150). The bottom panel shows TEM micrographs of the CTTP-50 and CTTP-150 samples at different magnifications.

Figure 1 shows conventional TEM images of pristine TNT, CTNT, and CTTP samples (CTTP-50 and CTTP-150) at different magnifications. These images provide a valuable insight into the effects of thermal treatment in an inert atmosphere on the size, shape, and occurrence of the TNTs, while also illustrating the influence of ANI content on the morphology of the resulting composites.

The TNTs used as precursors for the synthesis of TTP nanocomposites exhibited lengths of up to 100 nm, with an outer diameter of about 10 nm and an inner diameter of about 8 nm. Thermal treatment of the TNTs in an inert atmosphere significantly altered their original tubular morphology. As a result, the structure transformed predominantly into faceted nanoparticles (NPs), with a small fraction of TNTs and various nanostructures in the form of short rod-like shapes and elongated spheroids remaining.

The CTTP-50 sample synthesized with a higher ANI content exhibits well-defined hexagonal NPs with sharp edges, indicating a high degree of crystallinity. Residual tubular structures can still be observed in this sample, along with certain amorphous regions due to the formation of graphene-like carbonaceous domains from the carbonized PANI. Furthermore, the high ANI concentration likely enables the controlled growth of crystalline TiO_2_ domains by influencing nucleation and morphology during thermal processing.

The CTTP-150 sample synthesized with a lower ANI content shows the formation of a network-like structure along with a higher degree of particle agglomeration, suggesting that the lower amount of ANI limits the stabilization of individual particles during synthesis. In addition, graphene-like nanosheets derived from carbonized polyaniline are observed in this sample, further highlighting the structural changes induced by the thermal treatment.

These observations confirm that the amount of ANI used during synthesis plays an essential role in defining the morphology and distribution of graphitic carbon structures in the nanocomposites. Literature indicates that the synthesis parameters—including oxidant to monomer ratio, pH, and polymerization rate—have a significant impact on the resulting PANI chain length, oxidation state, and interfacial compatibility with TiO_2_, influencing both the structural evolution during carbonization and the final photocatalytic performance of the composite [13,18]. In this study, the aniline content was systematically controlled by adjusting the molar ratios of TiO_2_/ANI (150 and 50), which allowed precise tuning of the carbon layer thickness. The resulting differences were further investigated to evaluate their effects on the molecular and structural properties of the composites, as well as on their adsorption capacity and photocatalytic activity.

### 2.2. Molecular Structure

**Figure 2:**
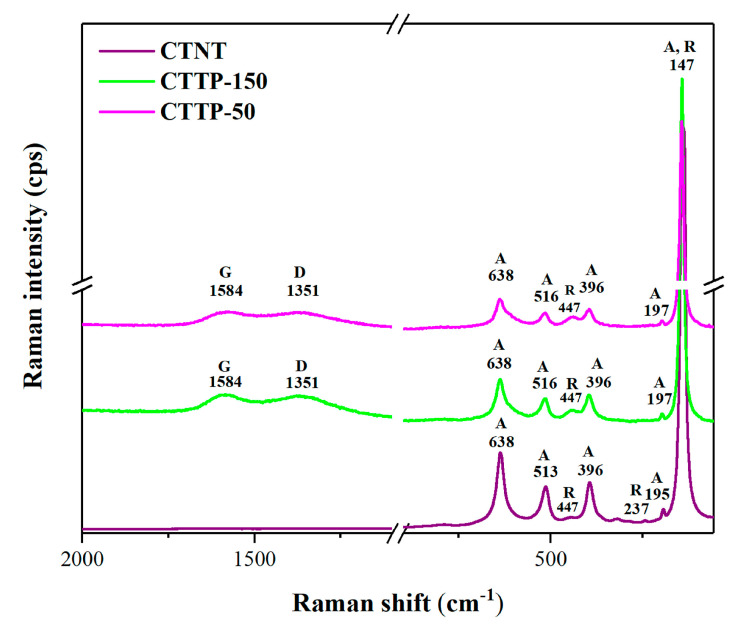
Raman spectra of pristine nanotubes, CTNT, and CTTP nanocomposites obtained after the carbonization process at 650 °C in an inert atmosphere.

Raman spectroscopy was used to investigate the structural changes in the nanocomposites and pristine TNT after thermal treatment at 650 °C in an inert atmosphere and to check whether the PANI part in the composites was completely carbonized (Figure 2). The Raman spectrum of annealed pristine TiO_2_-NTs (CTNT) shows a strong vibrational band at 147 cm^−1^, which can be associated with both the anatase and rutile crystal phases, bands at 195, 396, 513, and 638 cm^−1^, which are assigned exclusively to the anatase phase, and two weak bands at 237 and 447 cm^−1^, which are assigned to the rutile phase [12,13]. However, considering the significantly higher intensity of the anatase bands compared to the rutile bands and the absence of other characteristic rutile bands (the strongest rutile band at about 615 cm^−1^ is most likely masked by the much stronger anatase band at 638 cm^−1^), it can be concluded that carbonized pure TiO_2_ NTs (CTNT) are predominantly in anatase form.

In addition to the bands observed in the spectrum of CTNT, the CTTP nanocomposites (CTTP-50 and CTTP-150) exhibit additional broad bands centered at 1584 cm^−1^ and 1351 cm^−1^ originating from the carbonized PANI moiety in the composites [13,27,28,29]. The band at 1584 cm^−1^ corresponds to the G (graphitic) band, which originates from the E_2g_ phonon mode of the sp^2^-hybridized carbon atoms involved in the stretching vibrations within the aromatic rings and conjugated chains. The D (disorder-induced) band at 1351 cm^−1^ is attributed to breathing modes of sp^2^ carbon atoms in disordered six-membered rings, indicating the presence of structural defects [27,28,29]. The simultaneous presence of the D and G bands confirms the formation of disordered carbonaceous structures as a result of the decomposition of PANI and complete carbonization, which is confirmed by the absence of the characteristic bands.

In addition, the Raman spectra of CTTP-50 and CTTP-150 show the reappearance of bands associated with the anatase phase of TiO_2_, particularly at 147 cm^−1^ (E_g_), 197 cm⁻^1^ (E_g_), 396 cm⁻^1^, 516 cm⁻^1^ (B_1g_), and 638 cm⁻^1^ (E_g_) [12,13]. These results indicate that the carbonization of the composite material leads to a mixed-phase TiO_2_ structure in which anatase is partially retained or recrystallizes into the rutile crystalline phase. In particular, the 447 cm⁻^1^ band remains noticeable and is attributed to the rutile Eg mode, while the 147 cm⁻^1^ band likely represents a superposition of rutile B_1g_ and anatase E_g_ modes, reflecting the coexistence of both crystalline phases in the carbonized composite [13]. Similarly to CTNT, the Raman spectra indicate that the anatase phase dominates in the CTTP composites. Furthermore, the TiO_2_-derived bands are stronger in the spectrum of CTTP-150 compared to CTTP-50, as expected due to the higher initial amount of TiO_2_ in CTTP-150.

These results suggest that the presence of PANI in CTTP nanocomposites plays a key role during the carbonization process by preventing complete phase transformation, helping maintain the anatase crystal phase, and promoting the formation of carbonaceous regions in the composite [13].

### 2.3. Optical Properties and Band Structure Analysis

**Figure 3:**
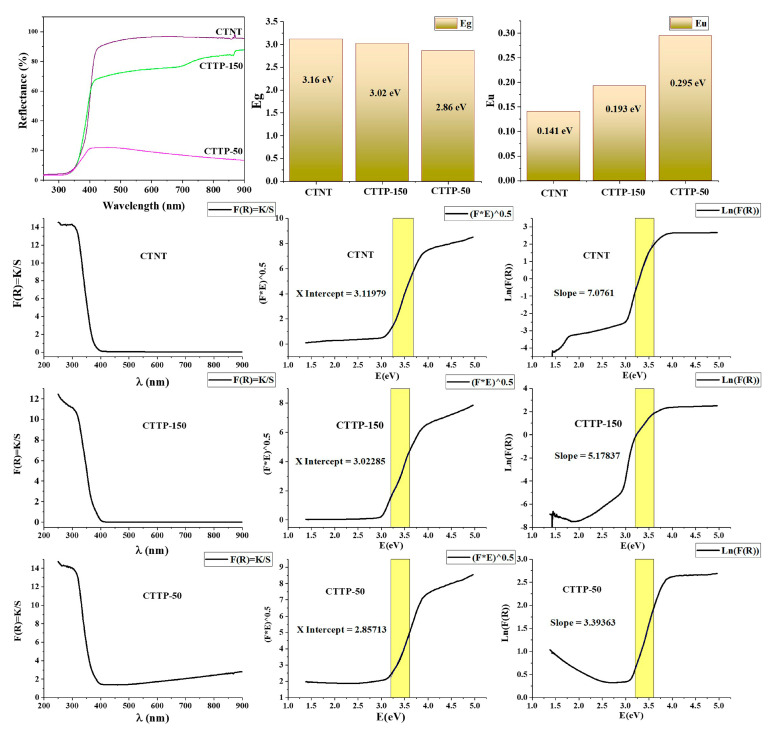
Comparative analysis of optical properties of CTNT and CTTP nanocomposites. The yellow rectangle marks the region used for curve linearization.In addition to the band gap (E_g_), the Urbach energy (E_u_) was also evaluated to assess the degree of structural disorder and the density of localized states within the band gap. The E_u_ values were extracted from the exponential absorption tail region by linearly fitting the ln[F(R)] against the photon energy (right column in Figure 3). The values obtained were 0.141 eV for CTNT, 0.193 eV for CTTP-150, and 0.295 eV for CTTP-50, indicating a progressive increase in electronic disorder at higher PANI content.

The optical properties and electronic band structure of the synthesized samples, CTNT and CTTP nanocomposites with different ANI content (CTTP-150 and CTTP-50), were thoroughly investigated by DRS, and the results are shown in Figure 3.

The original DRS spectra and the Kubelka–Munk transformed reflectance data (upper left panel in Figure 3) show a sharp absorption edge in the UV region for all samples. The CTNT sample shows an apparent decrease in reflectance from about 400 nm, indicating a band gap transition typical for mixed anatase/rutile TiO_2_. After the in situ polymerization of ANI and subsequent carbonization, an increasing red shift of the absorption edge is observed for CTTP-150, which is even more pronounced for CTTP-50 and indicates an increased absorption in the visible range. This shift is attributed to the possible incorporation of carbonaceous and nitrogenous species from the PANI framework into the TiO_2_ crystal lattice during thermal transformation. This transformation leads to the introduction of center-hole states in TiO_2_ [33] and extends the π-conjugation of PANI within the composite structure.

Tauc plots for indirect transitions were generated to estimate the optical band gap (E_g_) by plotting (F(R)hν)^1/2^ against the photon energy [34,35]. The extrapolated linear components yield E_g_ values of 3.16 eV (CTNT), 3.02 eV (CTTP-150), and 2.86 eV (CTTP-50), confirming a significant narrowing of the band gap with increasing PANI content and degree of carbonization. This trend indicates a successful tuning of the electronic structure of TiO_2_ through interfacial interaction with the CPANI matrix. In our case, the presence of Ti–C, Ti–N, and Ti–O–C/N bonds formed during carbonization likely introduces impurity states and oxygen-vacancy-like defects within the TiO_2_ band structure. These states bridge the valence and conduction bands and lower the excitation energy required for electronic transitions. Consequently, this narrowing of the band gap improves the absorption of visible light, which has a positive effect on the photocatalytic performance of the composites.

This systematic increase in Urbach energy across the CTTP sample series reflects a greater density of defect states, likely due to the incorporation of carbon and nitrogen species during PANI carbonization. Such defects—Ti^3+^ centers, sp^2^-hybridized carbon clusters, and nitrogen doping sites—disrupt the crystalline TiO_2_ network and create shallow, localized states within the band gap, manifesting as an extended Urbach tail. These properties are often observed in disordered semiconductor systems. For example, in studies of PANI films, a significant increase in Eu (from 0.57 eV to 2.24 eV) was found with film thickness and disorder, accompanied by a concomitant decrease in the bandgap [36]. Comparable results were reported in the study by Kolhar et al. [37]. Similarly, Choudhury et al. reported increased Eu values for oxygen-depleted, vacuum-annealed TiO_2_ and attributed them to the formation of defect-rich structures [38]. In our case, the increase in E_u_ values confirms the structural complexity generated by PANI-derived carbonaceous phases, which can facilitate absorption below the band gap and support enhanced charge separation properties beneficial for photocatalytic applications under visible light.

The optical study confirms that the integration of carbonized PANI with TiO_2_-NTs not only decreases the band gap but also increases the structural disorder, both of which should improve light absorption and enable better charge dynamics in photocatalytic applications.

### 2.4. Crystallographic Analysis

**Figure 4:**
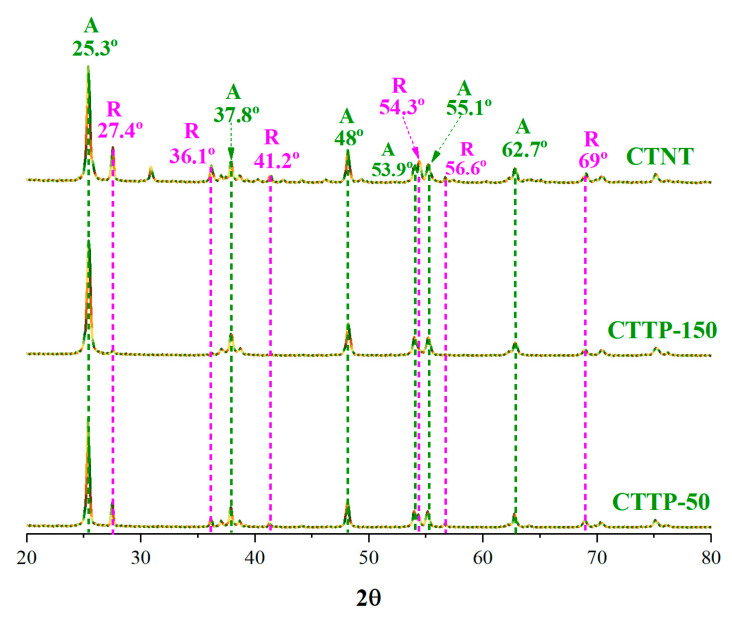
XRD patterns of CTNT and CTTP nanocomposites obtained after carbonization at 650 °C. The peaks of the anatase and rutile phases are denoted with A and R, respectively.

The crystallographic structure of all synthesized samples was investigated by XRD analysis, and the diffraction patterns obtained are shown in Figure 4.

All samples, both annealed pristine TiO_2_ NTs (CTNT) and the synthesized CTTP nanocomposites, show distinct diffraction peaks corresponding to the anatase and rutile phases of TiO_2_. The peaks obtained at 2θ = 25.3° (A101), 37.8° (A004), 48.0° (A200), 53.9° (A105), 55.1° (A211), and 62.2° (A204) can be assigned to the anatase phase (ICDD PDF 21-1272), while the peaks at 27.4° (R110), 36.1° (R101), 41.2° (R111), 54.3° (R211), 56.6° (R200), and 69.0° (R301) correspond to the rutile TiO_2_ (ICDD PDF 21-1276).

The absence of pronounced diffraction peaks corresponding to carbon (typically broad peaks around 24° and 44° for graphitic structures) [13] confirms a very low content of carbonized PANI, which is consistent with the low initial molar ratio of ANI/TiO_2_ used in the synthesis. The result obtained may also mean that the carbon derived from the carbonization of PANI is either amorphous or forms a very thin coating on the TiO_2_ surface due to its very low content, which is below the detection limit of the XRD technique.

The results obtained with the Spurr and Myers equation are shown in Table 1, and it can be seen that even after carbonization at 650 °C, the anatase phase predominates in all samples.

Figure 4 shows that the intensity ratio of the anatase (101) to rutile (110) peaks is much higher for CTTP-150 than for CTTP-50, indicating a stronger stabilization of the anatase phase when a lower initial concentration of polyaniline was used. This is also confirmed by the calculations shown in Table 1. It is noted that the percentage of the rutile phase in CTTP-150 (10.03%) is more than twice as low as its content in CTTP-50 (22.65%). This indicates that the single- or double-layer carbonized polyaniline structure (in CTTP-150) acts as a better protective agent during the carbonization process, by reducing the conversion of anatase to rutile, than the multilayer carbonaceous surface content (in CTTP-50). Similar results were observed in our previous study [13].

The presence of carbonaceous species could inhibit the transformation of anatase to rutile through the physical entrapment of TiO_2_ nanoparticles [13], which could be related to the observed morphological changes. The content of carbonized polyaniline and rutile was found to have the same direction of change; accordingly, some observations could be made. A lower content was associated with the presence of faceted morphology (CTTP-150 and CTNT), while a higher content was associated with a mixed morphology that included both residual nanotubes and newly formed faceted nanostructures such as nanorods and nanospheres. In addition, previous studies have shown that the degree of carbonization and the distribution of carbon on the surface of TiO_2_ NPs significantly affect the crystallization behavior, defect density, and electronic properties [39]. On the other hand, few reasons for anatase stabilization could be found in the literature, such as chemical bonds (e.g., Ti–C or Ti–N bonds) that stabilize the anatase structure [40,41], and the reduction of grain growth rate, which is a critical factor for phase transformation [42]. The content of morphological and phase changes correlates strongly with the results of the Debay–Scherrer equation.

The average crystallite size determined with this equation is shown in Table 1. These results show a similar trend to the TEM microscopy results (Figure 2) and the results of the Scherrer equation calculations (Table 1). The presence of a carbonized thin PANI layer on TiO_2_, especially in the CTTP-150 sample, contributed to a slight decrease in crystallite size compared to the carbonized pure TiO_2_ nanotubes (CTNT). The small difference between CTNT and CTTP-150 suggests that the carbonized PANI network effectively restricted crystal growth during annealing, likely by forming a physical barrier around the TiO_2_ grains and by chemical interaction with the TiO_2_ surface. This observation is consistent with previous studies showing that carbonaceous materials inhibit grain growth and stabilize the anatase phase during thermal treatment [13].

In contrast, the significantly larger crystallite size (~27 nm) observed for CTTP-50 suggests that a multilayer carbonaceous content on the TiO_2_ surface does not act as a protective agent. Due to different carbonization mechanisms, the mobility of the carbon network in multilayer carbon coatings is quite high, which promotes the mobility of grain boundaries and enables enhanced crystal growth during the carbonization process. Similar trends have been reported in studies where lower carbon content led to larger crystallite size in TiO_2_-based composites [43].

Smaller crystallites are generally associated with a higher density of grain boundaries, which can serve as active sites for photocatalytic or adsorption processes. Therefore, CTTP-150, with its combination of high anatase content and smaller crystallite size, is expected to have better functional performance than CTTP-50. This is consistent with findings highlighting the enhanced photocatalytic activity of anatase TiO_2_ with smaller crystallite sizes due to a larger surface area and active sites.

### 2.5. Thermal Analysis

**Figure 5:**
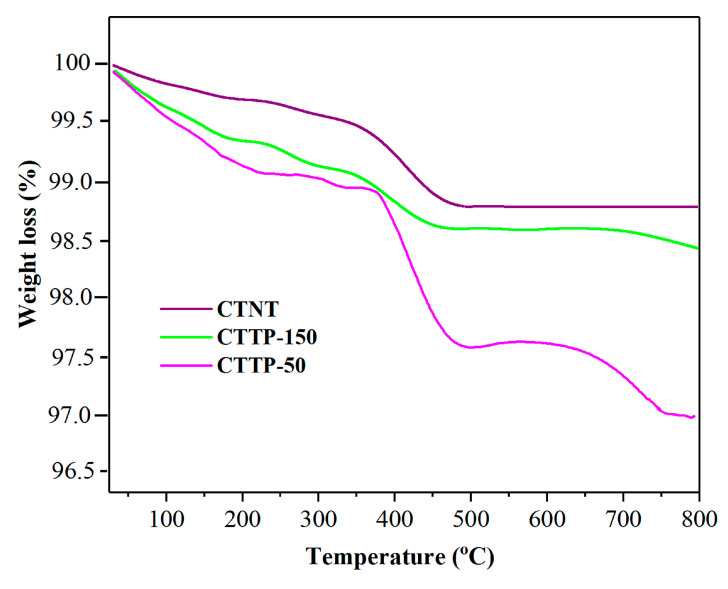
TGA analysis of CTNT and CTPP nanocomposites obtained after the carbonization process.

In this study, thermogravimetric analysis (TGA) was used with a focus on quantifying the amount of carbonized PANI in CTTP nanocomposites. In addition, the synthesized samples were analyzed by TGA to evaluate their thermal degradation behavior and gain a deeper insight into the underlying degradation mechanisms, as shown in Figure 5.

All synthesized samples exhibited an initial weight loss of less than 0.6% between 50 and 145 °C, which can be attributed to the dehydration process and the release of adsorbed water molecules from the sample surface. This observation is consistent with the typical evaporation of moisture trapped in the polymer matrix or only weakly bound to the polymer backbone.

A more pronounced weight loss occurred in the second degradation phase, which took place between 200 and 500 °C and corresponds to the decomposition of the polyaniline component in samples CTTP-50 and CTTP-150. This phase reflects the breakdown of the polymer chains under elevated thermal conditions. In addition, thermal degradation of the CTTP nanocomposites occurs when the polymer is exposed to temperatures above 500 °C, resulting in a weight loss of 1.5% and 3% for CTTP-150 and CTTP-50, respectively, determining the actual content of carbonized PANI in the final composites. These results are in full agreement with the initial experimental conditions, considering the initial molar ratio of ANI to TiO_2_.

Regarding the influence of TiO_2_ on the thermal stability of the synthesized CTTP nanocomposites, it can be observed (Figure 5) that the complete thermal decomposition of CTTP-150 starts at around 460 °C. In contrast, the complete decomposition of the CTTP-50 sample starts at about 500 °C, which confirms the positive effect of TiO_2_ on the thermal stability of the synthesized nanocomposites. Similar results have also been reported in the literature [44,45,46].

### 2.6. Adsorption Study

**Figure 6:**
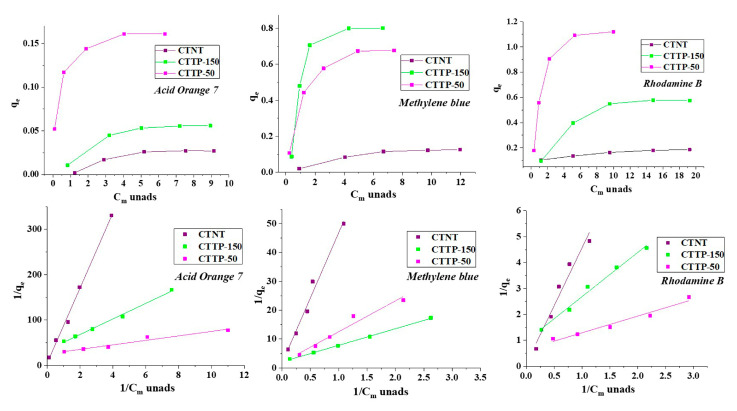
Adsorption isotherms of AO7, MB, and RB after 30 min equilibration in the dark.

The adsorption efficiency of the synthesized samples CTNT, CTTP-150, and CTTP-50 towards selected dye molecules (AO7, MB, and RB) was systematically investigated, and the results are shown in Figure 6. The obtained isotherms for all selected dyes closely follow the Langmuir adsorption model, suggesting a single-layer coverage of dye molecules on homogeneous adsorption sites. The adsorption capacity increased for all investigated nanocomposites regardless of the dye type, which further supports the Langmuir assumption of energetically equivalent binding sites.

The adsorption isotherms (upper panel) show a rapid increase in dye uptake during the initial adsorption phase (first 30 min), followed by a gradual plateau, indicating that equilibrium has been reached. This typical behavior indicates an initial abundance of available active sites, followed by progressive saturation as time progresses. The adsorption isotherms exhibit a characteristic L-shaped profile as classified by Charles et al. [47]. This shape indicates minimal competition between the dye molecules and the water for the adsorption sites on the catalyst surface [48,49]. Furthermore, this indicates that the dye molecules are likely localized at the solid–liquid interface, particularly in the interplanar regions of the catalyst, mainly through weak lateral interactions (Langmuir type).

The diagrams in the lower part of Figure 6 show the linear fit of the experimental data to the Langmuir adsorption model. The high degree of linearity observed in all cases confirms the suitability of the Langmuir model to describe the adsorption mechanism, which assumes a single-layer coverage of the dye molecules on a homogeneous distribution of active sites. The parameters of adsorption and photocatalytic profile obtained by using TNT and TPC nanocomposites for selected dyes (AO7, MB, RB) are shown in Table 2. The calculated Langmuir constants (Table 2) support the trend observed in the experimental data.

The maximum adsorption constant (K_ads_) derived from the Langmuir fit shows the following order: CTTP-150 > CTTP-50 > CTNT. This order mirrors the kinetic adsorption results and suggests that carbonized PANI improves surface affinity towards dyes, likely through π–π interactions between aromatic dye structures and conjugated carbon domains, as well as hydrogen bonding and electrostatic interactions facilitated by nitrogen-containing functionalities.

Of the materials studied, CTTP-150 exhibited the highest adsorption capacity for almost all three dyes and reached equilibrium faster and with greater overall uptake compared to CTTP-50 and CTNT. These results strongly suggest that a lower PANI content after carbonization leads to a more favorable surface architecture that improves the accessibility of the adsorption sites, as confirmed by TEM microscopy and XRD measurements (the smallest nanostructures and crystallite size were detected in CTTP-150). From a theoretical point of view, the enhanced performance of CTTP-150 can also be attributed to a higher degree of surface functionalization, including nitrogen-doped carbon domains introduced via carbonized PANI, providing additional active sites for interaction with dye molecules [50]. The moderate performance of CTTP-50 despite its higher PANI content may be explained by partial aggregation or pore-blocking effects during synthesis and carbonization, reducing the number of accessible active sites.

The lowest performance of CTNT, which consists solely of carbonized TiO_2_-NTs, is consistent with its relatively lower surface area, fewer functional groups, and lack of conductive polymeric carbonaceous extensions.

Due to its highest adsorption affinity among all selected dyes, CTTP-150 can be considered as a promising candidate with superior photocatalytic efficiency.

### 2.7. Photocatalytic Study

**Figure 7:**
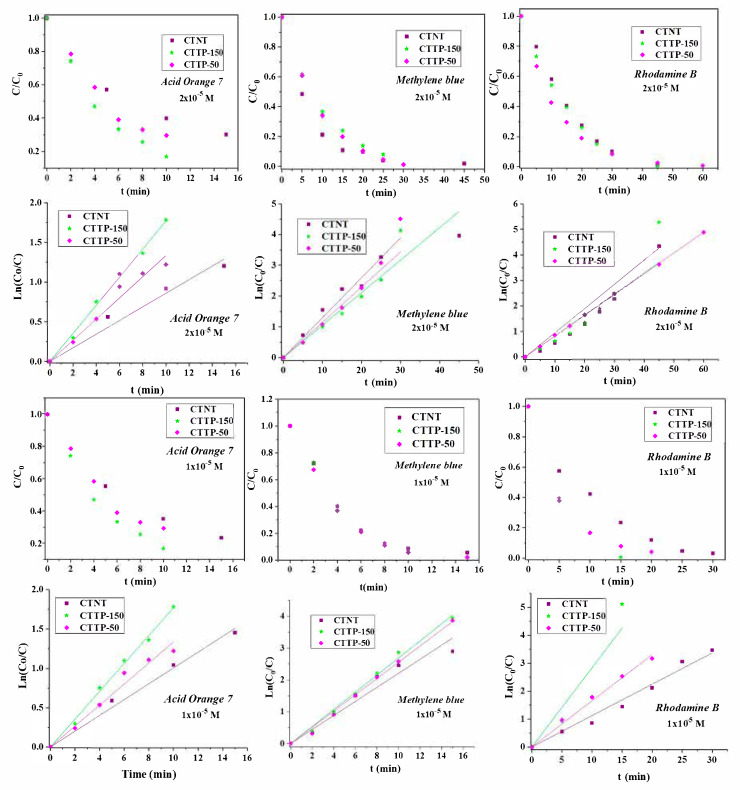
Photocatalytic activity of different concentrations (1 × 10^−5^ and 2 × 10^−5^ M) of AO7, MB, and RB in the presence of neat CTNT and CTTP nanocomposite samples (CTTP-150 and CTTP-50). All experiments were performed after 30 min dark equilibration to eliminate the contribution of initial dye adsorption; degradation data shown reflect only the photocatalytic effect under visible-light irradiation.

The photocatalytic degradation activity of CTNT, CTTP-150, and CTTP-50 samples was evaluated for three model organic dyes (AO7, MB, and RB) under visible-light irradiation. Prior to light irradiation, all dye dispersions were stirred in the dark for 30 min to achieve adsorption–desorption equilibrium. This ensured that the subsequent decrease in dye concentration during the photocatalytic tests (as shown in Figure 7) was due solely to photocatalytic degradation and not to initial adsorption. The contribution of adsorption was evaluated separately and is discussed in Section 2.6, allowing a clear distinction between physical adsorption and light-induced photodegradation.

As shown in Figure 7, all samples tested showed photocatalytic activity, with the efficiency varying depending on the type and concentration of the dye and the composite structure.

To investigate the influence of dye concentration on the photocatalytic performance of the tested material, degradation experiments were performed with two concentrations of each dye: 1 × 10^−5^ and 2 × 10^−5^ mol/L. These values correspond to the mass concentrations listed in Table 2 and Table 3. The selected concentrations were selected to be within the range used in the adsorption study (1, 3, 5, 7, and 9 mg/L). In this way, the photocatalytic experiments were preceded by an adsorption analysis carried out under comparable concentration conditions.

Complete decolorization of all dyes was achieved within 60 min for both concentrations tested. As shown in Figure 6 (first panel), degradation of the 2 × 10⁻^5^ M solutions of AO7, MB, and RB occurred after 15, 45, and 60 min, respectively. In contrast, at the lower concentration of 1 × 10⁻^5^ M (Figure 7, third panel), the degradation of AO7, MB, and RB was completed after 15, 30, and 30 min, respectively.

These results show that AO7 is degraded within the same time frame regardless of the initial concentration, while the degradation rate of MB and RB increases by approximately 33% and 50%, respectively, when the dye concentration is halved.

The linear correlation obtained from the graphs confirms that the photodegradation process follows pseudo-first-order kinetics. The calculated k values support the trend of photocatalytic activity for all three dyes: CTTP-150 > CTTP-50 > CTNT.

Among the tested samples, CTTP-150 consistently showed the highest photocatalytic performance for all three dyes, most likely due to the synergistic effect between the obtained TiO_2_ nanostructures and the carbonaceous matrix, both formed during the carbonization process of the synthesized materials. This composite structure allows for increased light absorption, improved charge separation, and more effective transfer of light-generated charge carriers. It has been reported that carbon modification of TiO_2_ can increase visible-light absorption and suppress charge recombination, thereby improving photocatalytic performance [51].

In addition, the morphology of the TiO_2_ nanotubes plays a non-negligible role in improving the overall performance of the nanocomposites. Their one-dimensional structure enables efficient and directional charge transport along the tube axis, reducing the recombination of charge carriers—a standard limitation in photocatalysis with conventional TiO_2_ particles. In addition, their hollow structure and large specific surface area provide accessible active sites for the adsorption of dye molecules and the generation of reactive oxygen species (ROS) under light irradiation.

These structural features also promote close contact between the semiconductor surface and the pollutant molecules, which is essential for efficient photodegradation. According to the findings of Wang et al., TiO_2_-NTs showed better photocatalytic activity compared to TiO_2_ nanoparticles due to improved light collection, reduced agglomeration, and increased electron mobility [52]. Similarly, Zhang et al. demonstrated that composites based on TiO_2_-NTs exhibited enhanced charge separation and higher degradation efficiency, especially when integrated into heterostructures, highlighting the advantages of nanotubular architecture [53].

These findings support our experimental observation that the presence of TiO_2_-NTs in the CTTP samples contributes significantly to their superior photocatalytic efficiency compared to results obtained in our previous work, where carbonized polyaniline-based nanocomposites and colloidal TiO_2_ nanoparticles were used [13]. This enhancement can be attributed to the unique properties of TiO_2_ nanotubes, which facilitate better charge separation and increased surface area, allowing for more active sites for photocatalytic reactions. The comparative analysis underscores the effectiveness of these new composites in degrading organic dyes under visible-light irradiation.

The knowledge gained from the optical and X-ray diffraction analyses contributes significantly to the understanding of the role of TiO_2_ in controlling the photocatalytic performance of the synthesized composites. As shown in the results presented in Figure 2 and Table 1, several structural and electronic features synergistically enhance the activity of the CTTP-150 sample. These include the presence of defect states and the possible formation of Ti–C and Ti–N bonds, which are known to reduce the band gap energy and promote visible-light absorption. Ren et al. confirmed that Ti^3^⁺ surface sites, nitrogen doping, and oxygen vacancies strongly affect the reduction of the band gap of TiO_2_, enabling better absorption in the visible spectrum and improving redox reaction kinetics [54]. In addition, the CTTP-150 sample exhibits an optimal anatase to rutile phase ratio (A/R) of about nine, which has been associated with improved photocatalytic performance due to the synergistic effect between the two TiO_2_ crystal phases. Similar results have been reported in the literature, confirming that the balanced mixture of anatase and rutile phases forms heterojunctions that enhance charge separation and suppress recombination, leading to higher activity under visible light [55]. In addition, the nanometer crystallite size provides a favorable balance between surface area and crystallinity, enabling efficient charge transport while minimizing recombination [56,57]. Taken together, these properties clearly position CTTP-150 as the most efficient photocatalyst among the tested materials, which is consistent with the results of photocatalytic degradation and kinetic studies.

Perhaps the most important factor contributing to the excellent photocatalytic performance of the CTTP-150 sample is the remarkably high adsorption constants, as shown in Table 2. These values indicate that this photocatalyst is able to effectively bind dye molecules to its surface, facilitating close contact between the pollutants and the reactive sites. This enhanced adsorption promotes interactions between photon-induced species and the dye molecules, i.e., reactive oxygen species (ROS) generated upon exposure to light can easily participate in redox reactions with the adsorbed dye molecules. As a result, the overall photocatalytic degradation process is significantly accelerated, emphasizing the dominant role of CTTP-150 in dye removal efficiency. These results are consistent with previous studies by Wang, Y. et al. indicating that strong dye adsorption improves the interaction between pollutants and ROS, accelerating the degradation processes and increasing the overall photocatalytic efficiency [58,59]. The CTTP-50 sample showed moderate activity, while the pristine CTNT sample exhibited the lowest degradation rates, highlighting the positive role of carbonized PANI at an optimal concentration.

This performance trend indicates that the presence of an optimal amount of carbonized PANI (as in CTTP-150) enhances photocatalytic activity more effectively than a higher amount (CTTP-50), while the absence of PANI-derived carbon structures (CTNT) results in lower reactivity. The observed differences can be attributed to the presence of reactive functional groups on the material surface and consequently to the adsorption capacity and charge carrier dynamics.

**Scheme 1:**
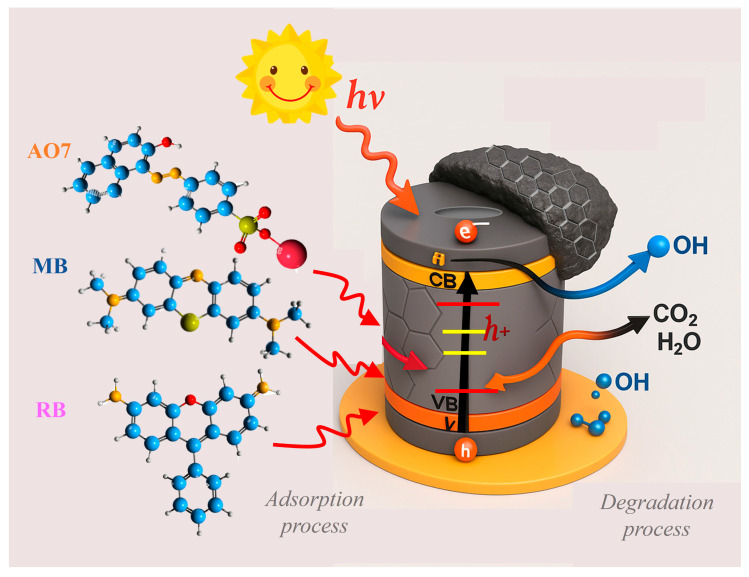
Schematic representation of the photocatalytic degradation mechanism of selected dyes on CTTP nanocomposites.

A schematic illustration of the proposed photocatalytic degradation mechanism of the selected dyes under sunlight irradiation is provided in Scheme 1.

When irradiated with visible light, the carbonized PANI/TiO_2_ NT nanocomposite absorbs photons and generates electron–hole pairs (e^−^/h⁺). This process can occur across a wide range of states within the band gap. Thus, various types of electronic states exist in the band gap of carbonized PANI/TiO_2_ nanocomposites, including lattice impurities caused by Ti–C, Ti–N, and Ti–O–C/N bonds, as well as local defect states such as Ti^3^⁺ centers and oxygen vacancies. Additionally, carbonized polyaniline contributes to π-conjugated carbon networks and nitrogen-doped domains that generate mid-gap states, enhance visible-light absorption, and promote charge delocalization and transfer, improving overall photocatalytic activity. Moreover, carbonized PANI, acting as a photosensitizer, enhances charge separation and suppresses recombination by facilitating the delocalization and transport of electrons. The excited electrons reduce the molecular oxygen adsorbed on the surface and form superoxide radicals (•O_2_^−^), which can further convert into hydroxyl radicals (•OH). Simultaneously, the holes in the valence band either participate directly in the oxidation of dye molecules or follow an alternative pathway by oxidizing H_2_O or OH- to create additional hydroxyl radicals (•OH). These reactive oxygen species attack the adsorbed dye molecules (R) and initiate successive oxidative degradation reactions. In the theoretical case, the end products are completely mineralized to harmless CO_2_ and H_2_O.

**Figure 8:**
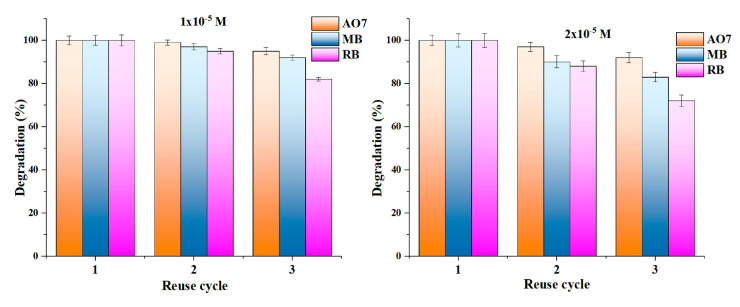
Multiple removal efficiency of CTTP-150 toward the investigated dyes (AO7, MB, and RB, concentrations 1 × 10^−5^ and 2 × 10^−5^ M) during three photodegradation cycles. Error bars represent standard deviation from three independent measurements, confirming the reusability and stability of the photocatalyst.

Considering the importance of the multiple uses of the photocatalyst, the sample with the best photocatalytic properties was selected (CTTP-150), and its reuse efficiency towards the studied dyes during three photodegradation cycles was investigated (Figure 8). It can be seen that the CTTP-150 sample shows high removal efficiency even after the second and third cycles for all dyes and both concentrations. The analysis shows improved reuse properties for the lower concentration used (1 × 10^−5^ M), which follows the photocatalytic degradation trend AO7 > MB > RB.

To evaluate the reproducibility of the photocatalyst performance, all reusability experiments were performed three times under identical conditions. The degradation efficiency values shown in Figure 8 contain error bars corresponding to the standard deviation of these repeated measurements. The minimal deviation observed across cycles demonstrates the excellent consistency and operational stability of CTTP-150 with repeated use.

Compared to previously reported PANI/TiO_2_ systems, the carbonized CTTP-150 nanocomposite developed in this study shows significantly improved photocatalytic and adsorptive performance. This improvement is due to the synergistic interplay between the nanotubular morphology of TiO_2_ and the nitrogen-doped carbon network formed during the carbonization of PANI. In contrast to conventional composites, CTTP-150 combines a reduced band gap, high anatase retention, and strong adsorption affinity, resulting in dye degradation of over 99% within 45 min after visible-light irradiation. These values exceed those reported for comparable systems in recent literature [19,20], demonstrating the advantage of our synthesis approach and material design for environmental remediation applications.

## 3. Materials and Methods

### 3.1. Materials

All chemicals used in this study were analytical reagent grade, purchased from Aldrich, and used as received without further purification.

### 3.2. Synthesis of Titania Nanotubes

The TNTs were synthesized using a hydrothermal method based on the procedure reported by Kasuga et al. [60], using commercial TiO_2_ powder (Fluka Chemie GmbH, Buchs, Switzerland) as starting material. Specifically, 2 g of TiO_2_ powder was dispersed in 50 mL of 10 M NaOH solution and hydrothermally treated in a Teflon-lined stainless steel autoclave (Parr acid digestion bomb, total capacity 125 mL) at 120 °C for 48 h under saturated water vapor pressure.

After the hydrothermal process, the resulting precipitate was thoroughly washed with 1 M HCl solution and then rinsed with distilled water. After each washing step, centrifugation was performed to separate the solid phase from the supernatant. This washing cycle was repeated until the pH of the supernatant reached a neutral value (pH ≈ 7). The final product was air-dried at 70 °C.

### 3.3. Synthesis of Carbonized PANI/TiO_2_ NT Nanocomposites

The synthesis of carbonized PANI/TiO_2_ NT (CTTP) nanocomposites was carried out in a two-step procedure. The first step for the synthesis of PANI/TiO_2_ NT nanocomposites (TTPs) involved the chemical oxidative polymerization of aniline (ANI) in the presence of TNT using ammonium peroxydisulfate (APS) as an oxidant according to established methods [12]. The process was carried out at room temperature with initial [TiO_2_]/[ANI] molar ratios of 50 and 150, with the resulting nanocomposites designated as TTP-50 and TTP-150, respectively. These molar ratios were chosen to systematically evaluate the influence of PANI content on the structure and performance of the resulting nanocomposites. The molar ratio of [APS]/[ANI] was fixed at 1.25.

For the synthesis, solutions of APS (0.025–0.1 M, 10 mL) and aniline (0.02–0.1 mM, 10 mL) were added simultaneously to a TNT suspension (0.2 M, 80 mL). Due to the relatively low ANI concentration, polymerization proceeded slowly, so that the reaction mixtures had to be stirred continuously for 24 h. To ensure the removal of residual monomers, oxidants, and low-molecular-weight by-products formed during polymerization, the nanocomposites were subjected to extensive dialysis with Milli-Q deionized water. After dialysis, the samples were dried in a vacuum at 60 °C until a constant mass was achieved.

**Scheme 2:**
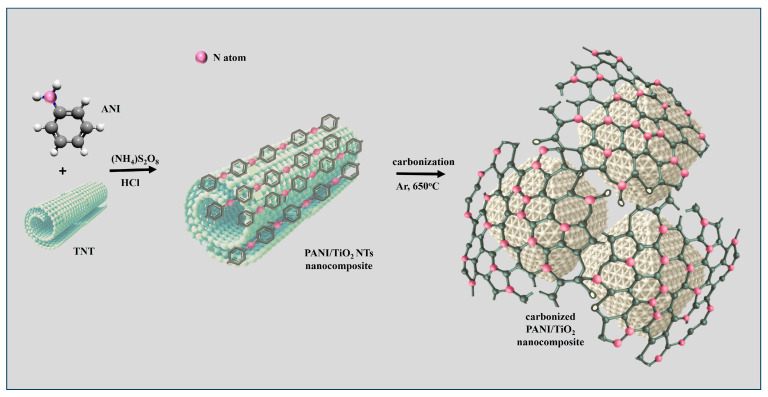
Illustration of the two-stage synthesis route used for the preparation of CTTP nanocomposites.

The second step consisted of thermal carbonization of the dried TTP nanocomposites under an argon atmosphere (Ar). The temperature was increased in a controlled manner from room temperature to 650 °C and maintained at 650 °C for 15 min, followed by a cooling phase to room temperature. Throughout the heating and cooling cycles, the samples were saturated in an Ar atmosphere for 30 min before and during the process. According to the predetermined [TNT]/[ANI] molar ratios, the CTTP nanocomposites were successfully synthesized following a previously described protocol. A schematic representation of the CTTP nanocomposite synthesis procedure is shown in Scheme 2. Pristine TNTs were also temperature-treated under the same conditions (650 °C, 15 min, inert (Ar) atmosphere), and the obtained powder was designated as CTNT and used as a reference sample.

### 3.4. Characterization

Transmission electron microscopy (TEM) of the TNT sample was performed using a JEOL JEM-1400 Plus electron microscope, JEOL Ltd., Tokyo, Japan with a voltage of 120 kV and a LaB6 filament. TEM imaging of the CTTP nanocomposites was performed using a Hitachi H-7000 FA TEM, Hitachi High-Technologies Corporation, Tokyo, Japan at 125 kV.

Raman spectra of the nanocomposite samples were collected using a Thermo Scientific DXR Raman microscope, Thermo Fisher Scientific, Waltham, MA, USA equipped with a HeNe gas laser with an excitation wavelength of 633 nm. The system included a research-grade optical microscope and a CCD detector. The laser beam with a power of 0.5 mW was focused with a 50× magnification objective onto the sample, which was placed on a motorized XY stage. The scattered light was analyzed with a spectrograph equipped with a 600-line per mm grating.

UV–Vis spectra were recorded with a Shimadzu UV2600 spectrophotometer, Shimadzu Corporation, Kyoto, Japan equipped with an ISR-2600 Plus integrating sphere.

The Kubelka–Munk function, commonly referred to as *F(R)*, is a theoretical model used to relate the reflectance of a diffusely scattering material to its absorption and scattering properties [34,35].

The function is defined as:

$$F(R)=\frac{{\left(1-R\right)}^{2}}{2R}$$

where R is the absolute reflectance (ratio of reflected to incident light) of the sample.

This function is proportional to the absorption coefficient (*K*) divided by the scattering coefficient (*S*) of the material:

$$F(R)=\frac{K}{S}$$

The X-ray powder diffractograms of the nanocomposites were recorded using a Philips PW 1050 powder diffractometer with Ni-filtered Cu Kα radiation (λ = 1.5418 Å). The data were recorded using the step scanning method with a step size of 0.05° and a counting time of 50 s per step.

The proportion of anatase and rutile phases in pure carbonized TiO_2_ and nanocomposites (CTTP-50 and CTTP-150) was determined from the area under the XRD peaks using Spurr and Myers’ equation [61]:

$$F_{R}=\frac{100}{1+1.26\left[A_{A}\left(101\right)/A_{R}\left(110\right)\right]}$$

where $F_{R}$ is the percentage of rutile and $A_{A}$(101) and $A_{R}\left(110\right)$ are the integral areas under the (101) anatase peak and the (110) rutile peak, respectively.

The constant 1.26 in Spurr and Myers’ equation is an empirical factor that applies when using the (101) peak of anatase and the (110) peak of rutile [61]. It takes into account the differences in scattering and peak intensities between the two phases. This value is not universal and may vary if different diffraction peaks or measurement conditions are used.

If the full width at half maximum (FWHM) of the anatase peak (101) is measured, the average crystallite size (*D*) can be estimated using the Debay–Scherrer equation [62]:

$$D=\frac{K\lambda }{\beta cos\theta }$$

where *D* is the average crystallite size (nm), *K* is the shape factor (~0.9), *λ* is the X-ray wavelength (Cu Kα = 0.15418 nm), *β* is the FWHM in radians, and *θ* is the Bragg angle.

Thermogravimetric analysis (TGA) was performed using a TGA701 Thermogravimetric Analyzer, LECO Corporation, St. Joseph, MI, USA in an oxygen atmosphere. Measurements were performed at a flow rate of 20 mL/min and a heating rate of 10 °C/min.

### 3.5. Adsorption Properties Study

To investigate the possible presence of several types of adsorption sites in the synthesized samples for the selected dye molecules (AO7, MB, and RB) and the influence of their chemical nature on the overall adsorption capacity, an adsorption study was performed. To investigate the adsorption behavior of selected dyes on the surfaces of the synthesized materials, untreated CTNT and CTTP-150 and CTTP-50 nanocomposites, equilibrium adsorption isotherms were recorded after 30 min of equilibrium in the dark (Figure 6), varying the dye concentration from 1 to 9 mg/mL.

The Langmuir model parameters were extracted by applying the least squares method to fit the linearized form of the Langmuir equation [63]:

$${\frac{1}{Q}}_{e}=\frac{1}{Q_{max}}+\frac{1}{K_{ads}Q_{max}C_{e}}$$

where *Q_e_* (mg/g) is the amount of adsorbed dye per unit mass of the observed sample at equilibrium, *Q_max_* (mg/g) is the theoretical maximum adsorption capacity, *C_e_* (mg/L) is the equilibrium concentration of unadsorbed dye in the solution, and *K_ads_* (L/mg) is the Langmuir adsorption constant under dark conditions, reflecting the affinity between the dye molecules and the adsorption sites.

The calculated values of *K_ads_* and *Q_max_* for all samples are summarized in Table 2.

### 3.6. Photocatalytic Ability Study

The photocatalytic performance of the synthesized samples was evaluated by monitoring the degradation of three dye molecules AO7, MB, and RB. Aqueous solutions of the dyes (2 × 10^−5^ and 1 × 10^−5^ mol/L) were prepared, and each solution was mixed with 1 g/L of either CTTP photocatalyst or annealed TNT.

The dispersions were continuously stirred and irradiated with sunlight from an Osram (Munich, Gernamy) Vitalux lamp (300 W) emitting white light with UVB (280–315 nm, 3.0 W radiant power), UVA (315–400 nm, 13.6 W radiant power), and visible and IR components. The optical power at a distance of 30 cm from the dispersion was approximately 30 mW/cm^2^, measured with an R-752 universal radiometer and a PH-30 DIGIRAD sensor (Poway, CA, USA).

Prior to illumination, the dispersions were stirred in the dark for 30 min to establish an adsorption–desorption equilibrium (zero point). Depending on the dye and catalyst structure and dye concentration, 1 mL of the dispersion was taken from the samples at 2–5 min intervals during the first 30–60 min of illumination until the photodegradation process was complete. The photocatalyst was separated from the solution by centrifugation at 12,000 rpm for 20 min. The absorbance changes of the dye molecules (RB and MB) in the supernatant were measured using a Thermo Scientific Evolution 600 spectrophotometer, Thermo Fisher Scientific, Waltham, MA, USA. Absorbance maxima were recorded at 484 nm for AO7, 554 nm for RB, and 664 nm for MB.

The kinetic data (Figure 6, right panels) were fitted using the pseudo-first-order model, expressed as:

$$\ln(\frac{C₀}{C})=k\cdot t,$$

where k is the apparent rate constant.

Reuse of the photocatalyst was tested by repeated application of the same samples in three fresh dye solution batches, each followed by illumination.

Generative artificial intelligence (GenAI ChatGPT) tools were used to generate parts of Scheme 1 and Scheme 2, and to support grammatical editing of the manuscript.

## 4. Conclusions

This work presents a comprehensive investigation of carbonized PANI/TiO_2_ NT-based nanocomposites, with a focus on their structural, optical, adsorptive, and photocatalytic properties. The materials were synthesized using different starting molar ratios of TiO_2_/ANI and analyzed by detailed physicochemical characterization.

X-ray diffraction (XRD) analysis confirmed the presence and partial retention of the anatase TiO_2_ phase in the CTTP samples, especially in CTTP-150, suggesting that the presence of PANI during carbonization plays a stabilizing role in preserving the original crystal structure of TiO_2_. Raman spectroscopy confirmed these results and revealed vibrational features associated with both components (TiO_2_ and carbonized polyaniline).

Optical characterization by UV-Vis-DRS showed a red shift of the absorption edge for the CTTP samples, indicating improved light harvesting in the visible region due to the change in electronic structure caused by the nitrogen-doped carbon domains formed during PANI carbonization. The band gap decreased from 3.16 eV (CTNT) to 2.86 eV (CTTP-50), while the Urbach energy increased up to 0.295 eV, indicating increased structural disorder and defect state density, which is beneficial for visible-light photocatalysis.

The adsorption capacity and photocatalytic degradability of the nanocomposites were evaluated using three model dyes: Acid Orange 7, Methylene Blue, and Rhodamine B. Langmuir isotherm fitting and kinetic analysis confirmed that the CTTP-150 sample exhibited the highest adsorption affinity and photocatalytic performance due to the synergistic contribution of several factors: the obtained anatase structure, the enhanced light absorption, and the increased surface activity provided by the PANI-derived carbonaceous network. The maximum adsorption capacities reached 1.00 mg/g (AO7), 0.28 mg/g (MB), and 0.11 mg/g (RB), with corresponding K_ads_ values of up to 5.14 L/mg. CTTP-150 also achieved over 99% degradation of RB within 45 min of visible-light irradiation.

In addition, CTTP-150 exhibited the highest anatase content (89.97%) and the smallest crystallite size (17.8 nm), both of which contributed to improved surface activity and charge separation. The photocatalytic efficiency remained stable over three reuse cycles, with the best reusability recorded for AO7 at 1 × 10^−5^ M.

Overall, the results show that the integration of TiO_2_ nanotubes and carbonized polyaniline leads to multifunctional nanocomposites with excellent photocatalytic and adsorptive properties. The combined use of structural, spectroscopic, and functional analyses provided deep insights into the mechanisms that determine their performance. These results underline the potential of such materials for the development of efficient visible-light-powered systems for environmental remediation and pollutant removal.

## Figures and Tables

**Table 1:** Anatase and rutile phase content (determined by Spurr and Myers equation), FWHM, and crystallite size of CTNT and CTTP nanocomposites.

Sample	Rutile (%)	Anatase (%)	FWHM (°)	Crystallite Size (nm)
**CTNT**	16.8	83.92	0.442	18.4
**CTTP-150**	10.03	89.97	0.457	17.8
**CTTP-50**	22.65	77.35	0.302	27.0

**Table 2:** Adsorption and photocatalytic profiles of TNT and CTTP nanocomposites toward AO7, MB, and RB.

Sample	Acid Orange 7 (AO7)				Methylene Blue (MB)				Rhodamine B (RB)			
	*q_max_*	*K_ads_*	*k_app_*		*q_max_*	*K_ads_*	*k_app_*		*q_max_*	*K_ads_*	*k_app_*	
			1 × 10^−5^ M (3.50 mg/L)	2 × 10^−5^ M (7.00 mg/L)			1 × 10^−5^ M (3.74 mg/L)	2 × 10^−5^ M (7.48 mg/L)			1 × 10^−5^ M (4.79 mg/L)	2 × 10^−5^ M (9.58 mg/L)
**CTNT**	0.01839	**0.06573**	0.10157	0.07937	0.32484	**0.03535**	0.21876	0.10547	0.1957	**0.02195**	0.10938	0.09482
**CTTP-150**	0.03938	**5.14055**	0.17188	0.17797	0.57774	**0.36513**	0.26564	0.11328	1.55003	**1.00022**	0.28126	0.11225
**CTTP-50**	0.02974	**1.95749**	0.13282	0.13013	0.44623	**0.17113**	0.25001	0.12501	1.02208	**0.57312**	0.16407	0.08118

**Table 3:** Photodegradation efficiency of pristine CTNT and CTTP nanocomposites toward AO7, MB, and RB.

Sample	Acid Orange 7 (AO7)				Methylene Blue (MB)				Rhodamine B (RB)			
	*t* (min)	*%*	*t* (min)	*%*	*t* (min)	*%*	*t* (min)	*%*	*t* (min)	*%*	*t* (min)	*%*
	1 × 10^−5^ M (3.50 mg/L)		2 × 10^−5^ M (7.00 mg/L)		1 × 10^−5^ M (3.74 mg/L)		2 × 10^−5^ M (7.48 mg/L)		1 × 10^−5^ M (4.79 mg/L)		2 × 10^−5^ M (9.58 mg/L)	
**CTNT**	10	**64.80**	10	**60.10**	15	**94.50**	30	**97.00**	15	**76.50**	45	**98.70**
**CTTP-150**	10	**83.20**	10	**83.20**	15	**97.90**	30	**98.40**	15	**99.40**	45	**99.49**
**CTTP-50**	10	**70.50**	10	**70.50**	15	**98.05**	30	**98.90**	15	**92.10**	45	**99.25**

## Data Availability

The data presented in this study are available on request from the corresponding author.
